# Emotional Intelligence in Portuguese Youth: Age and Gender Differences

**DOI:** 10.3390/jintelligence13040048

**Published:** 2025-04-15

**Authors:** Adelinda Araújo Candeias

**Affiliations:** School of Health and Human Development and Comprehensive Health Research Center, University of Evora, 7004-516 Evora, Portugal; aac@uevora.pt

**Keywords:** emotional intelligence, perceived emotional intelligence, Portuguese youth, age differences, gender differences, emotional development, EQ-i:YV

## Abstract

Emotional intelligence (EI) plays a pivotal role in youth development, influencing well-being, social adaptation, and academic success. This study aimed to assess age- and gender-related differences in perceived EI among Portuguese youth using the Bar-On Emotional Quotient Inventory: Youth Version (EQ-i:YV), a validated and widely applied tool. A sample of 931 students aged 8 to 16 from various regions of Portugal was evaluated across five EI domains: intrapersonal, interpersonal, stress management, adaptability, and general mood. The results show that emotional intelligence changes during adolescence, with clear age and gender differences. The data shows that as adolescents grow older, their perceived emotional intelligence (PEI) tends to decline, especially in adaptability and intrapersonal skills. While stress management and interpersonal abilities stay steady, they increasingly struggle with self-awareness and emotional regulation. Interpersonal skills remain the strongest, reflecting solid social abilities, while intrapersonal skills are the weakest, pointing to challenges with emotional insight. This means that while social connection and stress resilience hold up, adapting to change and managing emotions become harder with age. Gender differences also emerged, with girls demonstrating higher interpersonal skills and stress management in early adolescence, while boys reported better general mood in mid-adolescence. Despite these differences, no significant variations were found in the global EQi:YV scores. These results challenge the assumption of a linear increase in EI with age and emphasize the importance of a nuanced understanding of EI development. The study highlights the need for interventions that support emotional development throughout adolescence and targeted educational interventions tailored to the specific emotional competencies of different age and gender groups.

## 1. Introduction

The study of emotional intelligence (EI) has advanced significantly, with prominent models offering unique perspectives on its components. Mayer and Salovey’s (1997) ability model defines EI as a set of cognitive abilities related to processing emotional information, framing it as a distinct intelligence involving skills in perceiving, understanding, and managing emotions. This model emphasizes EI as a cognitive ability akin to traditional intelligence, focusing on skills that can be developed through practice and learning (Mayer and Salovey 1997; Ciarrochi et al. 2001).

In contrast, Goleman’s (1995) model includes broader competencies, such as self-regulation, motivation, and empathy, essential for effective interpersonal relationships and leadership. This framework has been particularly influential in organizational settings, where perceived EI is regarded as crucial for teamwork and management (Goleman 1995).

The Bar-On model combines both trait- and ability-based aspects, defining EI as a mix of perceived emotional, personal, and social competencies that promote psychological well-being and adaptation (Bar-On 2000). This mixed-method approach includes stable traits like self-regard and optimism, alongside self-perceived skills such as adaptability, stress management, well-being, and mental health (Fernández-Berrocal and Extremera 2016; Petrides et al. 2007). This duality enables a nuanced characterization of EI, bridging the gap between stable traits and learnable skills (Bar-On 2006; Bar-On and Parker 2000).

Perceived emotional intelligence is conceptualized as a form of self-development that evolves throughout childhood and adolescence, as proposed by Harter (2012). Harter identifies six developmental stages: very early childhood (ages 2–4), early to middle childhood (ages 5–7), middle to late childhood (ages 8–10), early adolescence (ages 11–13), middle adolescence (ages 14–16), and late adolescence (ages 17–19). Self-concept is framed as both a cognitive and social construct, evolving alongside normative cognitive changes. Consequently, self-perception and expressed behavior vary across developmental periods, shaping self-identity and influencing emotional intelligence.

Understanding how self-perception and emotional intelligence develop from childhood to adolescence is crucial for fostering the emotional and social growth of young individuals in formal and informal education (e.g., Candeias et al. 2024a, 2024b; CASEL 2012, 2013, 2016, 2020; Franco et al. 2017; Galindo et al. 2018; Goldberg et al. 2019; Gomez-Baya et al. 2016; Kickbusch 2012.)

Research indicates that emotional development follows distinct stages, with significant differences influenced by both age and gender. Studies by Harter (2012) reveal that self-perception evolves progressively, as individuals acquire new cognitive and emotional abilities at each stage, shaping their sense of self and emotional competencies.

In late childhood (ages 8 to 10), children develop more differentiated and realistic self-perception, becoming aware of their strengths and weaknesses in various domains such as academic competence, social acceptance, and physical abilities (Harter 2012). Emotional regulation remains underdeveloped, but children become better at recognizing basic emotions. Peer comparisons significantly influence self-esteem and self-concept, with emotional competence emerging in specific areas (Ferrándiz et al. 2012; Chamizo-Nieto et al. 2021).

In early adolescence (ages 11 to 13), self-awareness and self-reflection increase, allowing for more nuanced self-evaluations. Emotional understanding improves significantly, though emotional regulation may remain inconsistent due to hormonal and social transitions (Denham and Weissberg 2004; Cabello et al. 2016). Internal conflicts may arise as adolescents navigate conflicting aspects of their identity, balancing family expectations with social identity.

During mid-adolescence (ages 14 to 16), self-concept coherence and integration become more evident, as adolescents develop a more stable sense of identity and long-term goals. Emotional regulation becomes more refined, including strategies like cognitive reappraisal and problem-solving, fostering stronger interpersonal relationships (Singh and Thapa 2023; Cabello et al. 2016). Peer influence remains significant, yet family support continues to shape emotional and social competencies.

Studies suggest that emotional intelligence tends to increase with age, driven by cognitive and emotional maturation (Pfeifer and Peake 2012). Specific EI components, such as emotional understanding and regulation, often follow an inverted-U trajectory, peaking during mid-adolescence (Cabello et al. 2016; Bizai et al. 2016; Esnaola et al. 2017). In contrast, dimensions such as adaptability, interpersonal skills, and intrapersonal awareness may decline in older adolescents, reflecting increased self-doubt and emotional challenges typical of identity formation (Harter 2012; Bizai et al. 2016). These changes are associated with greater cognitive maturity and evolving social experiences (Singh and Thapa 2023; Gutiérrez-Cobo et al. 2017; Esnaola et al. 2017; Franco et al. 2017).

Gender differences also become more evident during adolescence, particularly in emotional perception and stress management. Girls tend to excel in perceiving and understanding emotions and in empathy, while boys often show greater adaptability and stress management skills, especially in middle and late adolescence (Cabello et al. 2016; Ferrándiz et al. 2012; Singh and Thapa 2023). Research also indicates that girls typically underestimate their emotional abilities, whereas boys tend to overestimate them, with girls demonstrating greater accuracy and self-awareness in emotional self-assessment (D’Amico and Geraci 2022).

Exploratory studies with Portuguese children and adolescents indicate that girls exhibit higher levels of empathy and emotional perception, while boys tend to perform better in stress management and adaptability (Pereira 2022; Cartaxo 2012; Candeias et al. 2011; Leal 2010). Age-related differences demonstrate that EI follows an ascending trajectory during childhood, peaking in mid-adolescence, and subsequently stabilizing or changing as maturation continues (Pólvora 2017; Rebelo 2012). These insights highlight the importance of considering both age and gender when studying EI to develop targeted interventions that support emotional and social competence in educational and clinical settings.

In summary, emotional development during childhood and adolescence is a critical process that directly impacts well-being, adaptability, resilience, and self-concept formation. Various theoretical models emphasize the role of EI at this developmental stage. Mayer and Salovey (1997) focus on cognitive abilities related to emotion processing, while Goleman (1995) highlights its impact on interpersonal relationships and personal success. Bar-On (2000) adds that EI encompasses socio-emotional competencies that enhance overall well-being.

In this context, perceived emotional intelligence (PEI) becomes fundamental as it reflects individuals’ subjective perceptions of their emotional competencies. Analyzing age and gender differences in PEI among children and adolescents can inform educational and clinical practices aimed at promoting healthy emotional development.

The main objective of this study was to analyze age and gender differences in PEI among Portuguese children and adolescents, aged between 8 and 16 years, aiming to identify patterns that could inform educational practices and clinical interventions. The specific objectives were:

- To examine age-related differences in PEI and its components (based on Bar-On’s model of EI) among children and adolescents, considering specific age groups based on psychological, educational, and pediatric criteria.
- To evaluate gender differences in PEI, investigating whether significant differences exist or whether similarities prevail between boys and girls.

These objectives aim to guide the design of EI assessment and intervention programs within educational and clinical contexts.

## 2. Materials and Methods

### 2.1. Participants

A total of 931 youth from all regions of Portugal participated in data collection. These participants were enrolled in compulsory education in regular classes, attending primary and lower secondary education levels (basic compulsory education). The Portuguese organization of formal education for children and youth mandates compulsory education until the age of 18 (Education System Basic Law 1986), organized in two main levels (basic—from 6 to 15 years old; and secondary—from 15 to 18 years old), based on the pediatric classification of developmental stages from the American Association of Pediatrics (Allen and Waterman 2024). The collected sample, from basic education, presents an age range spanning from 8 to 16 years. The mean age for the sample was 11.78 years (SD = 2.36) (Table 1).

With the aim of capturing developmental differences in emotional intelligence in children and adolescents, we considered the variable age, as well as creating a variable named age group. Age group was defined based on: (i) levels of basic education in Portugal (1st level between 6 and 10 years old; 2nd level between 10 and 12 years old; 3rd level between 13 and 15–16 years old, where the last year (age rank 15–16 years old) marks the vocational decision and choice of life project); (ii) the of self-development proposed by Harter (2012); and (iii) the pediatric guidelines from the American Association of Pediatrics (Allen and Waterman 2024). It could be characterized as follows.

Group 1: Late Childhood (ages 8–10)—In this group, corresponding to the 2nd sub-level of basic education in Portugal (3rd and 4th grade), children begin to develop a more differentiated and realistic self-perception. They start recognizing their strengths and weaknesses across various domains, such as academic competence, social acceptance, and physical abilities. Their self-concept becomes increasingly dependent on external feedback from parents, teachers, and peers. Emotionally, they show improvement in identifying basic emotions in themselves and others, but their ability to regulate these emotions is still emerging. Peer comparison becomes a significant factor influencing their self-esteem and overall self-concept.

Group 2: Early Adolescence (ages 10–12)—Early adolescence corresponds to the 2nd level of basic education in Portugal, or 5th and 6th grade. During this period, children experience the onset of puberty, marked by physical changes such as the development of secondary sexual characteristics. These changes often trigger heightened self-awareness and body image concerns. Cognitive development leads to greater reflection on personal identity, although thinking remains mostly concrete, with a tendency toward black-and-white perspectives. Adolescents become increasingly concerned with peer acceptance and may show signs of egocentrism, feeling that they are constantly being observed and judged by others. Additionally, there is a growing need for privacy and independence, sometimes resulting in boundary testing and occasional conflicts with parents.

Group 3: Middle Adolescence (ages 13–14)—This level aligns with the first two years of 3rd level (7th and 8th grade) basic education in Portugal. Adolescents at this age work on integrating different aspects of their identity, striving for coherence in their self-concept. They become more capable of abstract thinking, enabling them to consider multiple perspectives and engage in more complex self-evaluations. Emotional regulation improves, and they begin to adopt more advanced strategies, such as cognitive reappraisal. Peer influence is particularly strong, especially in areas related to behavior, appearance, and social activities. This is also a time when romantic relationships and exploration of sexual identity become more prominent. While adolescents increasingly seek autonomy from their families, parental support remains crucial in helping them navigate these changes.

Group 4: Beginning of Late Adolescence (ages 15–16)—This age group, also part of the 3rd level of basic education (9th grade), is characterized by greater stability in self-concept and identity and the exploration of vocational options and the decision about the life project. Adolescents at this stage gain clarity about their personal values and begin setting long-term goals. Their capacity for empathy and perspective-taking improves, facilitating stronger and more meaningful interpersonal relationships. Although their cognitive abilities allow for more abstract and future-oriented thinking, impulsivity can still influence their decision-making, as brain development—particularly in the frontal lobes—is not yet complete. While they spend more time with peers and less with family, parental guidance continues to play a vital role in supporting emotional and social development.

This classification highlights key physical, cognitive, emotional, and social milestones in each age group, and facilitates comparisons, allowing for identification of age-specific competencies and support of tailored interventions. This structure also reduces internal variability within groups, enhancing the clarity and interpretability of results. Studies like that of Denham and Weissberg (2004) support this approach, showing that emotional competencies vary significantly across developmental stages. The sample is divided into four age groups: 8–10 years, 11–12 years, 13–14 years, and 15–16 years, with an almost even gender distribution of 502 females and 442 males, as we describe in Table 2.

### 2.2. Materials

Emotional quotient inventory—EQ-i—was the first tool to assess perceived emotional intelligence to be published by a psychological test publisher (Bar-On 1997), as well as the first to undergo peer review in the *Buros Mental Measurement Yearbook* (Plake and Impara 1999), and it remains one of the most frequently used tools for measuring emotional–social intelligence (Bar-On 2004; Bru-Luna et al. 2021). Detailed information about its psychometric properties and development is available in the Bar-On EQ-i Technical Manual (Bar-On 1997).

The EQ-i test consists of 133 short-item statements measured on a 5-point Likert scale, ranging from “very seldom or not true of me” (1) to “very often true of me or true of me” (5). Suitable for individuals aged 17 and older, the instrument takes approximately 40 min to complete. Bar-On later developed a youth version, the Emotional Quotient Inventory: Youth Version (EQ-i:YV), by adapting 96 of the original 133 items for youth aged 7 to 18, ensuring the content was appropriate for younger respondents. The Bar-On Emotional Quotient Inventory: Youth Version (EQ-i:YV), developed by Bar-On and Parker (2000), is a comprehensive tool crafted to assess the social and emotional functioning of children and adolescents between the ages of 7 and 18. Structured into five core competency areas—intrapersonal, interpersonal, stress management, adaptability, and general mood—the inventory includes 17 competencies and a total of 60 self-report items. Each item is measured on a 4-point Likert-type scale (1 = never, 2 = sometimes, 3 = almost always, 4 = always), encouraging self-reflection and insight into youths’ emotional intelligence. The tool has undergone multiple validation studies, which provide theoretical and empirical support for the adequacy of its scale structure. Bar-On and Parker (2000) reported an internal consistency of 0.84 for the intrapersonal subscale and 0.89 for the total scale, reinforcing the reliability of this measure.

The EQ-i:YV has consistently demonstrated its effectiveness in assessing EI across various youth populations, with validation studies from multiple countries confirming its robust psychometric properties. In the U.S. and Canada, studies such as those by Keefer et al. (2013) report strong internal consistency, with Cronbach’s alpha values ranging from 0.76 to 0.87, highlighting its reliability in both educational and clinical settings (as previously proposed by Bar-On (2000)). This solid psychometric foundation has led to its widespread use, offering valuable insights into individual differences in EI in diverse contexts. In Spain, a significant large-scale validation involving more than 5.200 adolescents confirmed the EQ-i:YV’s structural coherence and reliability across different demographic groups, with subscale reliability ranging from 0.63 to 0.80 (Esnaola et al. 2017).

In Portugal, several studies have demonstrated the applicability and validity of the Portuguese adaptation of the EQ-i:YV in assessing emotional intelligence (EI) among children and adolescents (Candeias and Rebocho 2007; Candeias et al. 2008, 2011, 2013). With these successive studies the scale was translated and culturally adapted by following established guidelines to ensure semantic equivalence while preserving the original theoretical framework. This adaptation showed good psychometric properties, with an internal consistency index of 0.87 for the total scale and reliability coefficients ranging from 0.69 to 0.86 across subscales (Candeias et al. 2011). Specifically, Candeias et al. (2008) reported reliability indices for the subscales as follows: Adaptability (0.86), intrapersonal and positive impression (0.83), interpersonal (0.81), emotional expression (0.70), and stress management (0.69), confirming the instrument’s robustness for use with Portuguese youth. Additional findings showed dimension alphas between 0.70 and 0.83, with the stress management subscale reporting a coefficient of 0.60. The total scale maintained an internal consistency index of 0.87, with the five key factors explaining 50.6% of the total item variance (Candeias et al. 2011).

The Portuguese version retains the original structure, organized into the following five main competency dimensions, each with specific subcomponents.

I. Intrapersonal Competencies: This dimension assesses an individual’s emotional self-awareness and personal capabilities. It represents the ability to understand, express, and communicate one’s emotions and needs.

1. Self-Awareness: Recognizes and comprehends personal emotions, essential for emotional insight.
2. Assertiveness: Expresses feelings, beliefs, and thoughts constructively, defending one’s rights without aggression.
3. Self-Regard: Maintains respect and acceptance for oneself, supporting a healthy self-view.
4. Self-Actualization: Identifies and nurtures personal potential, pursuing growth;
5. Independence: Demonstrates self-control and autonomy in thought and action, fostering emotional independence.
*Related Items: 7, 17, 28, 31, 43, 53*
*Example Item: “I find it easy to talk about my feelings.”*

II. Interpersonal Competencies: This group focuses on understanding and appreciating others’ emotions, fostering cooperative and meaningful relationships.

6. Empathy: Recognizes, understands, and values others’ feelings, crucial for social connection.
7. Social Responsibility: Engages constructively within a group, reflecting cooperative behavior.
8. Interpersonal Relationships: Builds and maintains satisfying social bonds.
*Related Items: 2, 5, 10, 14, 20, 24, 36, 41, 45, 51, 55, 59*
*Example Item: “I understand how others feel.”*

III. Stress Management Competencies: Focused on self-regulation under pressure, this dimension assesses resilience in challenging situations, fostering calm responses rather than reactive behaviors.

9. Stress Tolerance: Manages stress effectively, maintaining composure through adversity.
10. Impulse Control: Regulates impulsive behaviors, promoting thoughtful responses.
*Related Items: 3, 6, 11, 15, 21, 26, 35, 39, 46, 49, 54, 58*
*Example Item: “I know how to stay calm.”*

IV. Adaptability Competencies: This dimension evaluates the capacity to navigate everyday challenges by solving problems, validating emotions, and adjusting to new contexts and situations.

11. Problem Solving: Identifies and applies solutions to challenges in daily life.
12. Reality Testing: Validates emotions and assesses realistic perspectives in situations
13. Flexibility: Adjusts behaviors and emotions to changing circumstances.
*Related Items: 12, 16, 22, 25, 30, 34, 38, 44, 48, 57*
*Example Item: “I easily understand new things.”*

V. General Mood Competencies: Reflecting emotional positivity, this dimension examines the tendency to maintain a happy and optimistic outlook, which supports resilience and motivation.

14. Happiness: Reflects satisfaction with oneself and life in general, associated with positive affect.
15. Optimism: Maintains a hopeful attitude, emphasizing the brighter side of life.
*Related Items: 1, 4, 9, 13, 19, 23, 29, 32, 37, 40, 47, 50, 56, 60*
*Example Item: “I like who I am.”*

The EQ-i:YV includes two additional scales to ensure the accuracy and reliability of responses: the positive impression subscale and the inconsistency index. The positive impression subscale measures the tendency to give socially desirable responses, indicating when someone tries to present themselves in an overly favorable light. An example item from this scale is “I think I am the best at everything I do” (related items: 8, 18, 27, 33, 42, 52). If the score on this scale is too high (above 12), the response is excluded from the final sample to maintain accuracy. The inconsistency index evaluates response consistency by comparing pairs of items and calculating the difference between their scores. If the absolute difference exceeds 10, it indicates inconsistent responses, suggesting engagement issues. For this study, only inventories with valid scores on both the positive impression subscale (≤12) and the inconsistency index (≤10) were considered for analysis. These checks help maintain the reliability and validity of the results, minimizing response bias and ensuring meaningful assessment.

Based on factor analysis studies for the Portuguese population, we considered the raw totals for each subscale in this study (interpersonal, intrapersonal, stress management, adaptability, and general mood). To calculate the emotional intelligence quotient (EQ), the raw scores of the subscales are summed.

Demographic Questionnaire: The demographic questionnaire collected essential participant information, including age, gender, region and grade level.

### 2.3. Procedures

This study represents an extension and deepening of the RED—School Performance and Development project, a longitudinal initiative investigating the effects of educational transitions among Portuguese students in terms of development (social, emotional, cognitive), and school achievement (performance in standard tests of maths, language, and sciences and attitudes toward maths, language, and sciences) (PTDC/CPE-CED/104884/2008). Coordinated by the University of Évora and funded by the Portuguese Foundation to Science and Technology (Fundação para a Ciência e Tecnologia (FCT)), the RED project established a strong foundation for further investigation (some examples of previous studies: Vilia and Candeias 2019; Vilia et al. 2017). This current study builds upon the initial RED findings, and benefits from early studies about translation and validation of EQi:YV with specific samples, allowing for a more comprehensive and massive study of age and gender differences in perceived emotional intelligence, based on the EQi:YV for Portuguese Youth, providing the groundwork for an increasingly robust background for educational and clinical intervention.

To conduct the study, we obtained the necessary authorizations from the Portuguese Data Protection Commission (CNPD), and the Committee for Monitorization of Studies in Scholar Context from Ministry of Education—Directorate-General for Innovation and Curricular Development (MIME) with the number 0449600001, and the directors of participating schools and institutions. These approvals facilitated seamless collaboration with schools and guardians, enabling us to contact parents, who provided consent for their children’s participation. Only students with explicit parental informed consent were included.

Data collection was carried out by a team of researchers who visited schools previously selected across Portugal, from the north to the south, utilizing a convenience sampling approach that excluded all schools that took part in the RED project sample. Protocols were administered in group sessions of approximately 30 min each. The sample was collected between January and April 2023. Participants and their legal representatives were thoroughly informed about the study’s purpose, confidentiality, and anonymity protocols, and their right to withdraw at any time without penalty. This transparency reinforced participants’ trust and adherence to ethical standards.

This structured approach enabled precise and reliable data processing, analyzed with SPSS version 28.0 for Windows, allowing for an expanded and refined exploration of the insights gathered in earlier phases.

## 3. Analisys and Findings

### 3.1. Data Analysis

The data analysis was organized to address the study’s main objectives: (i) examine age-related differences in PEI and its components, and (ii) evaluate gender differences in PEI and its components.

The analysis of the results considered the scores from the total scale and the five subscales. The positive impression scale and the inconsistency index were not considered in the analyses. For the descriptive analysis, we used the sum of raw scores for each scale and subscale, to examine the minimum, maximum, mean, standard deviation, and the range of medium group raw punctuation for each scale and subscale across age groups. Such analysis was complemented by a graphical analysis based on the arithmetic means for each scale and subscale. In subsequent analyses, we relied on the raw scores for each scale and subscale.

Pearson’s correlation coefficients were considered to investigate the strength and direction of associations between age and the PEI dimensions. This step provided initial insights into whether age was significantly related to key aspects of perceived emotional intelligence and its dimensions.

The next step was the analysis of independent sample t-tests to assess gender differences in PEI and their dimensions. This analysis aimed to determine whether significant differences existed between male and female participants in overall PEI scores and each of their subcomponents.

To enhance the depth of the analysis, an analysis of covariance (ANCOVA) was performed. The primary goal of the ANCOVA was to explore the relationship between age and gender in relation to PEI. Age, considered a critical developmental variable, was included as a covariate to control for its potential confounding effects. By isolating the specific impact of gender while accounting for age, this approach provided a more nuanced understanding of how these two factors jointly influence perceived emotional intelligence. This method, guided by the methodological framework outlined by Tabachnick and Fidell (2007), enabled a robust exploration of the interplay between age, gender, and PEI among Portuguese children and adolescents.

Overall, this multi-step analytical strategy ensured a comprehensive examination of the research questions, offering valuable insights into developmental and gender-related patterns in perceived emotional intelligence.

### 3.2. Results

The analysis of descriptive statistics, based on raw scores for each total and subscale of the EQ-i:YV, revealed variations across different age groups (Table 3). These results suggest trends that must be interpreted with caution, as they are based on raw subscale scores, which are not directly comparable due to differences in the number of items per subscale.

Nonetheless, the data indicate that perceived emotional intelligence (PEI) tends to decrease with age, particularly in adaptability and interpersonal skills.

The youngest group (7–10 years) shows the highest total PEI score, with a mean of 140.26 (medium range: 123.78–156.74), suggesting positive emotional functioning. In the 11–12 years group, the total score slightly decreases to 138.51, possibly reflecting early emotional challenges along with the development of better coping mechanisms. This decline becomes more evident in the 13–14 years group, with a total score of 134.09, suggesting that this period may be particularly demanding from an emotional and social standpoint. The 15–16 years group presents the lowest total score (133.45), reflecting a gradual decline in PEI with age.

To enable a valid understanding between subscales with differing item counts, raw scores were transformed into mean scores per subscale (i.e., total scores divided by the number of items), resulting in values ranging from 1 to 4, with the theoretical midpoint at 2. This transformation allows for direct comparison across all dimensions, as we could observe in the graph.

**Figure 1:**
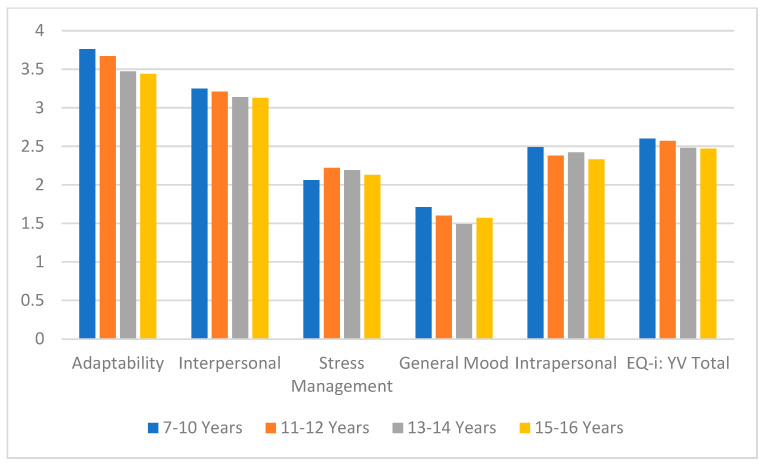
EQi:YV and subscales weighted averages (Min = 1, Max = 4).

Figure 1 presents these mean scores across age groups and confirms a progressive decline in overall emotional intelligence with age. Among the subscales, adaptability consistently shows the highest mean scores, reflecting a relatively strong perceived ability to manage change and adjust to new demands. In contrast, general mood displays the lowest mean values across all age groups, suggesting a decline in optimism and emotional well-being over time. This pattern supports the notion that self-perception and emotional awareness may be areas of greater difficulty in this age range.

Correlation analysis supports these findings, revealing a significant negative correlation between PEI and age (*p* < .01) (Table 4). This suggests that as adolescents grow older, there is a gradual decline in their perceived intrapersonal and interpersonal competencies, adaptability, and general mood. These results highlight adolescence as an especially challenging and critical developmental period in terms of emotional self-perception and adjustment.

Following the correlational analysis, independent samples t-tests were conducted to assess gender differences in PEI and its dimensions.

The analysis of gender differences in perceived emotional intelligence across age groups, assessed through the subscales adaptability, interpersonal, stress management, general mood, intrapersonal, and EQ-i:YV total, revealed interesting patterns across different age groups. Independent-samples t-tests were conducted to explore how boys and girls perceived their emotional skills at various developmental stages. While differences in overall EI scores were less consistent, notable gender-based trends emerged in specific subscales (Table 5).

For 7–10-year-olds, no significant gender differences were observed in any subscale or the total EI score. Boys and girls reported similar perceptions of adaptability (t(332) = −1.218, *p* = .224), interpersonal skills (t(332) = 1.961, *p* = .051), stress management, general mood, and intrapersonal skills. This absence of significant differences suggests that, during this early developmental stage, both genders perceive their emotional and social competencies similarly.

Among 11–12-year-olds, initial gender differences began to appear. Girls reported higher perceived competence in interpersonal skills (t(218) = 3.467, *p* < .001) and stress management (t(218) = 3.029, *p* = .003), while no significant differences were found in adaptability, general mood, or intrapersonal skills. The total EI score also did not show significant differences (t(218) = 1.455, *p* = .147). This suggests that during early adolescence, girls perceive themselves as more socially competent and better at managing stress, but these differences do not yet translate into overall EI disparities.

In the 13–14-year-old group, gender differences became more evident. Girls consistently rated themselves higher in interpersonal skills (t(249) = 7.338, *p* < .001) and in stress management (t(249) = 3.545, *p* < .001). Boys rated higher in general mood (t(249) = −2.068, *p* = .040). These data suggested that this period of adolescence presents pronounced gender-based differences in perceived social and emotional skills.

Finally, in the 15–16-year-old group, gender differences diminished. Boys rated themselves higher only in general mood (t(124) = −2.890, *p* = .005), with no significant differences in other subscales or the total EI score. This indicates that, by late adolescence, boys and girls tend to perceive their emotional competencies more similarly, apart from boys’ increased perception of emotional well-being reflected in their general mood scores.

These findings suggest that while some age-specific gender trends in perceived emotional intelligence can be observed, there is no consistent, continuous gender effect across all age groups and EI dimensions. Gender differences appear to fluctuate depending on the developmental stage, with more pronounced differences observed during the 11–14-year-old period, particularly in the interpersonal skills and stress management subscales. However, by late adolescence, these differences seem to diminish, leading to a more balanced perception of emotional competencies between boys and girls.

Given these fluctuating trends, it remains unclear whether the observed gender differences reflect stable patterns or are influenced by transient developmental and social factors. This calls for further investigation using more sophisticated statistical techniques, such as analysis of covariance (ANCOVA), to control for potential confounding variables and better clarify the nature of these differences. To address this limitation, an ANCOVA was conducted, treating sex as the independent variable and age as a covariate. This approach allowed for a deeper understanding of how gender and age influence perceived EI while controlling for the natural developmental progression of emotional intelligence across childhood and adolescence (Table 6).

Age, a key developmental factor, was included as a covariate to isolate the specific effects of gender. This approach clarified trends, such as girls excelling in interpersonal skills across most groups and boys scoring higher in stress management and general mood in older groups. ANCOVA also allowed for examining interaction effects between age and gender, offering a more nuanced understanding of EI perceptions. This method provided a clearer picture of how age and gender independently and jointly shape EI during childhood and adolescence (Tabachnick and Fidell 2007).

This analysis was chosen because age, a continuous variable, plays a developmental role in shaping perceptions of emotional and social competencies. By controlling for age, the model allows for a clearer examination of potential differences between males and females while accounting for the natural progression of emotional intelligence perceptions with age.

For the adaptability subscale, which measures individuals’ perceived ability to adjust to new situations, age emerged as a significant predictor (F(1, 926) = 44.719, *p* < .001, partial η^2^ = 0.046). However, sex did not significantly influence these perceptions (F(1, 926) = 1.758, *p* = .185, partial η^2^ = 0.002), indicating that both males and females perceived their adaptability similarly.

On the interpersonal subscale, which assesses self-perceived social and relational skills, both age and sex had significant effects. Age showed a small yet meaningful influence (F(1, 926) = 14.068, *p* < .001, partial η^2^ = 0.015), while sex had a stronger impact (F(1, 926) = 49.606, *p* < .001, partial η^2^ = 0.062). Females reported higher levels of perceived interpersonal skills compared to males, with a noticeable gender gap highlighted by these results.

Regarding the stress management subscale, which reflects individuals’ perceived capacity to handle stress effectively, age significantly influenced perceptions (F(1, 926) = 21.019, *p* < .001, partial η^2^ = 0.022), showing that older participants felt more capable of managing stress. Sex also had a significant but smaller, effect (F(1, 926) = 9.189, *p* = .003, partial η^2^ = 0.010), with females reporting slightly higher confidence in their stress management abilities compared to males.

For the general mood subscale, which gauges individuals’ perceptions of their overall positivity and optimism, age was a strong predictor (F(1, 926) = 78.394, *p* < .001, partial η^2^ = 0.090), accounting for 9% of the variance. Sex also had a smaller but significant effect (F(1, 926) = 13.937, *p* < .001, partial η^2^ = 0.017), with males reporting slightly higher perceptions of general mood compared to females.

On the intrapersonal subscale, which evaluates self-awareness and emotional regulation, age again showed a significant effect, though smaller compared to other subscales (F(1, 926) = 7.614, *p* = .006, partial η^2^ = 0.008). However, sex did not significantly influence these perceptions (F(1, 926) = 1.225, *p* = .269, partial η^2^ = 0.001), indicating similar levels of perceived intrapersonal skills across genders.

For the total emotional intelligence score, age had a significant effect (F(1, 926) = 26.513, *p* < .001, partial η^2^ = 0.028). On the other hand, sex did not have a significant influence on the total EI score (F(1, 926) = 1.931, *p* = .165, partial η^2^ = 0.002), indicating that when age is controlled, males and females report similar levels of total perceived EI.

The data analysis reveals that perceived emotional intelligence (PEI) tends to decrease as young people grow older, with a particular decline in adaptability and intrapersonal skills. In contrast, interpersonal and stress management abilities remain relatively stable throughout adolescence. Among all age groups, interpersonal skills consistently scored the highest, reflecting strong social and relational abilities, while intrapersonal skills showed the lowest scores, highlighting ongoing challenges with self-awareness and emotional management. This pattern suggests that while adolescents generally maintain good social interaction and stress management skills, they tend to struggle more with adapting to change and understanding their own emotions. Consequently, the overall EQ-i:YV score gradually declines with age, primarily driven by reductions in adaptability and intrapersonal competencies. The ANCOVA results further revealed that age consistently influences perceptions of emotional intelligence, while gender differences are less consistent and vary by subscale. Notably, gender differences were significant only on the interpersonal and stress management subscales. Although both age and gender contributed to some variance in perceived EI, the relatively low partial η^2^ values indicate that other factors, such as socio-environmental influences and personality traits, may significantly shape emotional intelligence perceptions.

## 4. Discussion

This study set out to explore age- and gender-related differences in perceived emotional intelligence (PEI) among Portuguese youth, examining how these differences evolve from late childhood to adolescence. Using Bar-On’s model, PEI was assessed across five key dimensions—intrapersonal, interpersonal, stress management, adaptability, and general mood—as well as a global PEI score. Bar-On’s framework emphasizes the importance of personal and social emotional competencies as fundamental components of well-being and successful adaptation. The results provide valuable insights into how emotional intelligence develops during adolescence, revealing clear age and gender-related patterns in self-perceived emotional competencies, as previous studies suggested (for example, Cartaxo 2012; D’Amico and Geraci 2022; Pereira 2022). The findings consistently show that interpersonal skills achieve the highest scores across all age groups, suggesting that young people maintain strong social and relational abilities throughout adolescence. On the other hand, intrapersonal skills consistently score the lowest, highlighting ongoing challenges with self-awareness and emotional management. This pattern suggests that while adolescents generally sustain good social interaction and stress management skills as they grow older, they tend to find it increasingly difficult to adapt to change and understand their own emotions. Consequently, the overall EQ-i:YV score gradually decreases with age, mainly driven by the decline in adaptability and intrapersonal competencies. These results align with previous studies that emphasize how adolescence can complicate emotional self-regulation despite stable social interaction skills (Gomez-Baya et al. 2016; Esnaola et al. 2017).

Interestingly, significant gender differences emerged primarily in the interpersonal and stress management subscales. During early adolescence, girls reported higher levels of perceived interpersonal skills and stress management, aligning with previous research that highlights girls’ early development of social awareness and empathy (Cabello et al. 2016; Ferrándiz et al. 2012). As adolescents progress into mid-adolescence, boys reported higher self-perceptions in stress management and general mood, which aligns with patterns observed in various cultural contexts (Chamizo-Nieto et al. 2021; Keefer et al. 2013). However, no significant gender differences were found in the global PEI scores, supporting previous literature (Meshkat and Nejati 2017) that suggests emotional intelligence levels tend to balance out when considering overall scores.

Clear age-related differences also emerged in stress management and general mood, with older youth reporting higher levels of perceived competence. These findings are consistent with previous studies (Chamizo-Nieto et al. 2021; Gomez-Baya et al. 2016; Ferrándiz et al. 2012), indicating that exposure to complex social situations and accumulated life experiences help adolescents build greater emotional resilience and better impulse regulation in high-pressure contexts. Effective stress management becomes increasingly important as adolescents navigate the emotional demands of both academic and social environments (Candeias et al. 2011, 2013; Franco et al. 2017).

However, an opposite trend was observed in adaptability, interpersonal skills, and intrapersonal awareness, where older adolescents reported lower self-perceptions. This decline challenges earlier assumptions that emotional intelligence increases linearly with age (Bar-On 2000; Bar-On and Parker 2000) and instead aligns with more recent findings (Esnaola et al. 2017; Bizai et al. 2016; Gomez-Baya et al. 2016), suggesting that adolescence, characterized by identity formation and heightened self-reflection (Harter 2012), may lead to increased self-doubt and emotional challenges.

### Limitations and Future Directions

Despite its valuable contributions, this study has limitations that must be considered. First, the cross-sectional design limits the ability to establish causal relationships or track changes over time. Although we used the positive impression scale and the inconsistency index, these measures did not control all the biases or inconsistencies in the participants’ responses related to self-reported measures like the EQ-i:YV, potentially affecting data accuracy.

The study’s focus on Portuguese youth also limits the generalizability of the findings to other cultural contexts. Although the sample size was considerable, it was drawn from a convenience sample of Portuguese schools, which may limit representativeness. Moreover, the study primarily focused on age and gender differences, overlooking other relevant variables such as family environment, academic performance, or social support, which might significantly impact emotional intelligence. Including these factors would provide a more nuanced analysis. Additionally, although the EQ-i:YV is a widely used and validated instrument, it primarily measures perceived emotional intelligence rather than actual abilities. So, future research, needs to guaranteed multitrait–multimethod measures, complementing self-reported information with objective measures of cognitive, social and neuroimaging data (Gutiérrez-Cobo et al. 2017; Pfeifer and Peake 2012), or multi-rater assessments that enhance the depth and reliability of the analysis (Petrides et al. 2007).

The relatively low partial η^2^ values observed in the ANCOVA analyses also indicate that other unmeasured factors, such as personality traits, cognition or social influences, might significantly shape perceived emotional intelligence. Future studies should address these additional variables to gain a deeper understanding of emotional development, and emotional intelligence construct.

Lastly, gender differences in PEI were significant only in certain subscales and were not consistent across age groups. This variability indicates that gender differences in emotional intelligence may be more nuanced and context-dependent than initially perceived, potentially influenced by developmental, social, or contextual factors (as previously suggested by Bar-On (2000)). Addressing these nuances through more targeted research would help clarify how gender and age interact in shaping emotional competence.

To conclude, this study underscores the importance of adopting a developmental and context-sensitive approach to understanding emotional intelligence. The improvements observed in stress management and general mood with age suggest that interventions focusing on these competencies in older adolescents could have positive outcomes. Conversely, the decline in perceived adaptability, interpersonal skills, and intrapersonal awareness during adolescence emphasizes the need for targeted support to develop these areas.

Given the importance of social–emotional competencies for well-being (Fernández-Berrocal and Extremera 2016), it is crucial to develop targeted interventions that address these vulnerabilities. Programs that focus on enhancing self-awareness and adaptability, while reinforcing social connection and stress management, can better support adolescents during this critical developmental phase (CASEL 2012, 2013, 2016, 2020; Chamizo-Nieto et al. 2021; Cristóvão et al. 2017, 2020). Moreover, future research should include longitudinal studies to capture changes over time and explore contextual factors such as cultural influences and socioeconomic backgrounds (Candeias et al. 2024a, 2024b; Goldberg et al. 2019; Galindo et al. 2018). Future intervention models should consider cultural influences as recommended by Bar-On (2000) and Candeias et al. (2011, 2025), by creating culturally adapted programs that resonate with the social realities faced by youth in diverse contexts. Holistic, multi-dimensional approaches involving youth, educators, families, and community stakeholders are essential to fostering emotional and social well-being across developmental stages. This integrated perspective not only promotes resilience and adaptability but also supports balanced socioemotional growth as youth transition.

## Figures and Tables

**Table 1:** Demographics by gender and age.

School Grade	N	(%)	Gender		Age	
			Female	Male	M	SD
3rd	44	4.7	21	23	8.77	0.097
4th	230	24.4	127	103	9.31	0.060
5th	58	6.1	32	26	9.36	0.166
6th	158	16.7	76	82	11.26	0.083
7th	117	12.4	63	54	12.47	0.063
8th	158	17%	90	60	13.91	0.721
9th	166	17.8%	86	80	14.81	0.783
Total	931	100.0	502	442	11.78	2.362

**Table 2:** Demographics by age group.

Age Group	N	Min	Max	(%)	M(SD)	Gender	
						Female	Male
1 (8–10)	334	7	10	35.9	9.12 (0.822)	179	155
2 (11–12)	220	11	12	23.6	11.65 (0.479)	114	106
3 (13–14)	251	13	14	27.0	13.64 (0.481)	147	104
4 (15–16)	126	15	16	13.5	15.33 (0.473)	55	71
Total	931	7	16	100.0	11.78 (2.36)	495	436

**Table 3:** Descriptive analysis for EQ-i:YV and subscales by age group.

Age Group	Scale/Subscale	N	Minimum	Maximum	Mean	Standard Deviation	Range of Medium Group
7–10 years old	Adaptability	334	20.00	52.00	37.59	6.02	31.57–43.61
	Interpersonal	334	16.00	44.00	38.97	4.58	34.39–43.55
	Stress Management	334	9.00	36.00	24.74	6.17	18.57–30.91
	General Mood	334	8.00	28.00	23.99	3.52	20.47–27.51
	Intrapersonal	334	6.00	24.00	14.96	3.66	11.3–18.62
	EQ-i:YV Total	334	80.00	178.00	140.26	16.48	123.78–156.74
11–12 years old	Adaptability	220	22.00	51.00	36.65	5.73	30.92–42.38
	Interpersonal	220	21.00	44.00	38.57	4.25	34.32–42.82
	Stress Management	220	15.00	36.00	26.66	4.37	22.29–31.03
	General Mood	220	10.00	28.00	22.38	3.80	18.58–26.18
	Intrapersonal	220	6.00	24.00	14.25	3.27	10.98–17.52
	EQ-i:YV Total	220	98.00	176.00	138.51	13.78	124.73–152.29
13–14 years old	Adaptability	251	16.00	48.00	34.69	5.29	29.4–39.98
	Interpersonal	251	26.00	44.00	37.72	4.34	33.38–42.06
	Stress Management	251	13.00	35.00	26.31	3.78	22.53–30.09
	General Mood	251	8.00	28.00	20.88	3.98	16.9–24.86
	Intrapersonal	251	6.00	24.00	14.50	2.99	11.51–17.49
	EQ-i:YV Total	251	96.00	176.00	134.09	12.75	121.34–146.84
15–16 years old	Adaptability	126	13.00	51.00	34.35	5.33	29.02–39.68
	Interpersonal	126	11.00	44.00	37.58	4.36	33.22–41.94
	Stress Management	126	16.00	36.00	25.56	3.92	21.64–29.48
	General Mood	126	10.00	28.00	22.01	3.81	18.2–25.82
	Intrapersonal	126	6.00	23.00	13.96	3.02	10.94–16.98
	EQ-i:YV Total	126	82.00	159.00	133.45	12.49	120.96–145.94

**Table 4:** Correlations between subscales and EQ-i:YV and age.

Sub-Scale	All Participants (N = 931)
Adaptability	−0.222 **
Interpersonal	−0.134 **
Stress Management	0.088 **
General Mood	−0.266 **
Intrapersonal	−0.090 **
EQ-i:YV Total	−0.192 **

** The correlation is significant at the 0.01 level (two-tailed).

**Table 5:** Gender differences in EQ-i:YV and subscales across age groups (N = 931).

Age Group	Subscale	Gender	N	M	SD	gl	T	Sig.
7–10 years old	Adaptability	Female	179	37.22	6.23	332	−1.218	0.224
		Male	155	38.02	5.75	332		
	Interpersonal	Female	179	39.43	4.21	332	1.961	0.051
		Male	155	38.45	4.94	332		
	Stress Management	Female	179	24.85	6.16	332	0.332	0.740
		Male	155	24.62	6.19	332		
	General Mood	Female	179	23.69	3.70	332	−1.688	0.092
		Male	155	24.34	3.26	332		
	Intrapersonal	Female	179	14.75	3.51	332	−1.120	0.263
		Male	155	15.20	3.82	332		
	EQ-i:YV Total	Female	179	139.94	17.10	332	−0.384	0.701
		Male	155	140.63	15.79	332		
11–12 years old	Adaptability	Female	114	36.35	5.82	218	−0.783	0.434
		Male	106	36.96	5.63	218		
	Interpersonal	Female	114	39.52	3.71	218	3.467	<0.001 *
		Male	106	37.56	4.57	218		
	Stress Management	Female	114	27.51	4.40	218	3.029	0.003 *
		Male	106	25.75	4.17	218		
	General Mood	Female	114	22.09	3.95	218	−1.194	0.234
		Male	106	22.70	3.61	218		
	Intrapersonal	Female	114	14.35	3.34	218	0.472	0.638
		Male	106	14.14	3.20	218		
	EQ-i:YV Total	Female	114	139.81	13.53	218	1.455	0.147
		Male	106	137.11	13.96	218		
13–14 years old	Adaptability	Female	147	34.89	5.33	249	0.697	0.487
		Male	104	34.42	5.25	249		
	Interpersonal	Female	147	39.25	3.71	249	7.338	<0.001 *
		Male	104	35.55	4.24	249		
	Stress Management	Female	147	27.00	3.53	249	3.545	<0.001 *
		Male	104	25.32	3.90	249		
	General Mxd esood	Female	147	20.44	3.99	249	−2.068	0.040 *
		Male	104	21.49	3.92	249		
	Intrapersonal	Female	147	14.51	3.27	249	0.098	0.922
		Male	104	14.47	2.55	249		
	EQ-i:YV Total	Female	147	136.09	12.98	249	3.012	0.003 *
		Male	104	131.25	11.91	249		
15–16 years old	Adaptability	Female	55	33.57	4.25	124	−1.510	0.134
		Male	71	34.95	5.99	124		
	Interpersonal	Female	55	38.39	3.67	124	1.854	0.066
		Male	71	36.95	4.76	124		
	Stress Management	Female	55	25.69	3.87	124	0.342	0.733
		Male	71	25.45	3.98	124		
	General Mood	Female	55	20.93	3.86	124	−2.890	0.005 *
		Male	71	22.85	3.58	124		
	Intrapersonal	Female	55	13.37	2.80	124	−1.922	0.057
		Male	71	14.41	3.13	124		
	EQ-i:YV Total	Female	55	131.95	10.84	124	−1.184	0.239
		Male	71	134.61	13.60	124		

* *p* < .05.

**Table 6:** ANCOVA results for gender and age effects on EQ-i:YV and subscales (N = 931).

Subscale	Source	Sum of Squares	df	Mean Squares	F	*p*	Partial η^2^
Adaptability	Age	1445.193	1	1445.193	44.719	<.001	0.046
	Sex	56.485	1	56.485	1.758	.185	0.002
	Error	29,744.289	926	32.121			
Interpersonal	Age	260.276	1	260.276	14.068	<.001	0.015
	Sex	916.503	1	916.503	49.606	<.001	0.062
	Error	17,108.536	926	18.476			
Stress Management	Age	502.850	1	502.850	21.019	<.001	0.022
	Sex	219.412	1	219.412	9.189	.003	0.010
	Error	22,110.999	926	23.878			
General Mood	Age	1111.707	1	1111.707	78.394	<.001	0.090
	Sex	193.494	1	193.494	13.937	<.001	0.017
	Error	12,856.222	926	13.884			
Intrapersonal	Age	83.691	1	83.691	7.614	.006	0.008
	Sex	13.435	1	13.435	1.225	.269	0.001
	Error	10,159.147	926	10.971			
Total	Age	5541.775	1	5541.775	26.513	<.001	0.028
	Sex	399.804	1	399.804	1.931	.165	0.002
	Error	191,757.108	926	207.081			

## Data Availability

Data are unavailable due to privacy or ethical restrictions.

## References

[B1-jintelligence-13-00048] Allen Britanny, Waterman Helen (2024). Stages of Adolescence.

[B3-jintelligence-13-00048] Bar-On Reuven (1997). The Emotional Quotient Inventory (EQ-i:YV): Technical Manual.

[B4-jintelligence-13-00048] Bar-On Reuven, Bar-On Reuven, Parker James D. A. (2000). Emotional and Social Intelligence: Insights from the Emotional Quotient Inventory (EQ-i:YV). Handbook of Emotional Intelligence.

[B5-jintelligence-13-00048] Bar-On Reuven, Geher Glenn (2004). The Bar-On Emotional Quotient Inventory (EQ-i): Rationale, Description, and Summary of Psychometric Properties. Measuring Emotional Intelligence: Common Ground and Controversy.

[B6-jintelligence-13-00048] Bar-On Reuven (2006). The Bar-On Model of Emotional-Social Intelligence (ESI). Psicothema.

[B2-jintelligence-13-00048] Bar-On Reuven, Parker James (2000). The Bar-On Emotional Quotient Inventory: Youth Version (EQ-i:YV) Technical Manual.

[B7-jintelligence-13-00048] Bizai Eugénia, Melo Madalena, Candeias Adelinda A., Veiga Feliciano H. (2016). Competências emocionais e comportamentos agressivos entre pares em estudantes de 2º e 3º ciclos de escolaridade. Student Engagement in School: Perspectives from Psychology and Education—Motivation for Academic Performance.

[B8-jintelligence-13-00048] Bru-Luna Lluna, Martí-Vilar Manuel, Merino-Soto César, Cervera-Santiago José (2021). Emotional Intelligence Measures: A Systematic Review. Healthcare.

[B9-jintelligence-13-00048] Cabello Rosario, Sorrel Miguel A., Fernández-Pinto Irene, Extremera Natalio, Fernández-Berrocal Pablo (2016). Age and Gender Differences in Ability Emotional Intelligence in Adults: A Cross-Sectional Study. Developmental Psychology.

[B10-jintelligence-13-00048] Candeias Adelinda, Félix Adriana, Dumitrache Anisoara, Almășan Beatrice, Gencel Ilke Evin, Zadworna Magdalena, Kossakowska Karolina, Sakellariou Angeliki, Sakellariou Miltos, Sahin Cavus (2025). Enhancing socio-emotional learning and mental health through computational thinking: A cross-cultural analysis of the COMPUSEL programme. Frontiers in Psychology.

[B11-jintelligence-13-00048] Candeias Adelinda, Rebocho Mónica (2007). Questionário de Inteligência Emocional de Bar-On para Crianças e Jovens—Tradução Portuguesa [Bar-On Emotional Intelligence Questionnaire for Children and Youth—Portuguese Translation].

[B12-jintelligence-13-00048] Candeias Adelinda, Portelada António, Félix Adriana, Galindo Edgar (2024a). Well-being and sustainability: Impact of teacher centred coaching model. International Journal of Innovation Science.

[B13-jintelligence-13-00048] Candeias Adelinda, Varelas Diana, Rebelo Nicole, Diniz António (2013). Validade Estrutural do Questionário de Inteligência Emocional: Estudo com Alunos do Ensino Básico Português [Structural Validity of the Emotional Intelligence Questionnaire: A Study with Portuguese Primary School Students]. Paper presented at I Seminar of Project RED—Rendimento Escolar e Desenvolvimento.

[B14-jintelligence-13-00048] Candeias Adelinda, Galindo Edgar, Reschke Konrad, Bidzan Mariola, Stueck Marcus (2024b). Editorial: The Interplay of Stress, Health, and Well-Being: Unraveling the Psychological and Physiological Processes. Frontiers in Psychology.

[B15-jintelligence-13-00048] Candeias Adelinda, Rebocho Mónica, Pires Heldemerina, Franco Glória, Barahona Helena, Charrua Marisa, Oliveira Manuela, Beja Maria João, Noronha A., Machado C., Almeida L., Gonçalves M., Martins S., Ramalho V. (2008). Estudos de Desenvolvimento da Prova de Competências Sociais: Socialmente em Ação (SA—360º). [Development Studies of the Social Skills Assessment: Socially in Action (SA—360º)]. Proceedings of the XIII International Conference on Psychological Assessment: Forms and Contexts.

[B16-jintelligence-13-00048] Candeias Adelinda, Rebelo Nicole, Silva João, Cartaxo Ana (2011). Bar-On—Inventário de Quociente Emocional (Bar-On EQ-i:YV): Estudos Portugueses com Crianças e Jovens do Ensino Básico [Portuguese Studies with Children and Youth in Primary Education.]. Paper presented at VIII Ibero-American Congress on Psychological Assessment.

[B17-jintelligence-13-00048] Cartaxo Ana (2012). Inteligência Emocional, Autoconceito e Bem-Estar Psicológico: Estudo Exploratório em Alunos de 9º, 10º e 11º Ano [Emotional Intelligence, Self-Concept, and Psychological Well-being: Exploratory Study in 9th, 10th, and 11th-Grade Students]. Master’s thesis.

[B18-jintelligence-13-00048] Chamizo-Nieto Maria Teresa, Arrivillaga Christiane, Rey Lourdes, Extremera Natalio (2021). The Role of Emotional Intelligence, the Teacher-Student Relationship, and Flourishing on Academic Performance in Adolescents: A Moderated Mediation Study. Frontiers in Psychology.

[B19-jintelligence-13-00048] Ciarrochi Joseph V., Chan Amy Y. C., Caputi Peter (2001). A Critical Evaluation of the Emotional Intelligence Construct. Personality and Individual Differences.

[B20-jintelligence-13-00048] Collaborative for Academic, Social, Emotional Learning (CASEL) (2012). Effective Social and Emotional Learning Programs: Preschool and Elementary School Edition.

[B21-jintelligence-13-00048] Collaborative for Academic, Social, Emotional Learning (CASEL) (2013). Implementing Systemic District and School Social and Emotional Learning.

[B22-jintelligence-13-00048] Collaborative for Academic, Social, Emotional Learning (CASEL) (2016). SEL Impact. http://www.casel.org/impact/.

[B23-jintelligence-13-00048] Collaborative for Academic, Social, Emotional Learning (CASEL) (2020). What Is SEL?. https://casel.org/what-is-sel/.

[B24-jintelligence-13-00048] Cristóvão Ana, Candeias Adelinda, Verdasca José (2017). Social and emotional learning and academic achievement in Portuguese schools: A bibliometric study. Frontiers in Psychology.

[B25-jintelligence-13-00048] Cristóvão Ana, Candeias Adelinda, Verdasca José (2020). Development of Socio-Emotional and Creative Skills in Primary Education: Teachers’ Perceptions About the Gulbenkian XXI School Learning Communities Project. Frontiers in Education.

[B26-jintelligence-13-00048] D’Amico Antonella, Geraci Alessandro (2022). Sex Differences in Emotional and Meta-Emotional Intelligence in Pre-Adolescents and Adolescents. Acta Psychologica.

[B27-jintelligence-13-00048] Denham Susanne A., Weissberg Roger P., Chesebrough Elda, King Patricia, Bloom Martin, Gullotta Thomas P. (2004). Social-Emotional Learning in Early Childhood: What We Know and Where to Go from Here. A Blueprint for the Promotion of Prosocial Behavior in Early Childhood.

[B28-jintelligence-13-00048] Education System Basic Law [Lei de Bases do Sistema Educativo] (1986). Diário da República n.º 237/1986, Série I de 1986-10-14. https://diariodarepublica.pt/dr/legislacao-consolidada/lei/1986-34444975.

[B29-jintelligence-13-00048] Esnaola Igor, Revuelta Lorena, Ros Iker, Sarasa Marta (2017). Desarrollo de la Inteligencia Emocional en la Adolescencia: Un Estudio Transversal y Longitudinal. Anales de Psicología/Annals of Psychology.

[B30-jintelligence-13-00048] Fernández-Berrocal Pablo, Extremera Natalio (2016). Ability emotional intelligence, depression, and well-being. Emotion Review.

[B31-jintelligence-13-00048] Ferrándiz Carmén, Hernández-Torrano Daniel, Bermejo Rosario, Ferrando Mercedes, Gómez Marta Sainz (2012). Social and Emotional Intelligence in Childhood and Adolescence: Spanish Validation of a Measurement Instrument. Revista de Psicodidáctica.

[B32-jintelligence-13-00048] Franco Maria G., Beja Maria J., Candeias Adelinda, Santos Natália (2017). Emotion Understanding, Social Competence, and School Achievement in Children from Primary School in Portugal. Frontiers in Psychology.

[B33-jintelligence-13-00048] Galindo Edgar, Candeias Adelinda A., Pires Heldemerina S., Carbonero Miguel (2018). School Achievement and Failure in Portuguese and Spanish Speaking Countries.

[B34-jintelligence-13-00048] Goldberg Jochem, Sklad Marcin, Elfrink Teuntje, Schreurs Karlein, Bohlmeijer Ernst T., Clarke Aleisha (2019). Effectiveness of interventions adopting a whole school approach to enhancing social and emotional development: A meta-analysis. European Journal of Psychology of Education.

[B35-jintelligence-13-00048] Goleman Daniel (1995). Emotional Intelligence: Why It Can Matter More Than IQ.

[B36-jintelligence-13-00048] Gomez-Baya Diego, Mendoza Ramon, Paino Susana (2016). Emotional Basis of Gender Differences in Adolescent Self-Esteem. Psicologia: Revista da Associação Portuguesa Psicologia.

[B37-jintelligence-13-00048] Gutiérrez-Cobo Maria Jose, Cabello Rosario, Fernández-Berrocal Pablo (2017). Performance-based ability emotional intelligence benefits working memory capacity during performance on hot tasks. Scientific Reports.

[B38-jintelligence-13-00048] Harter Susan (2012). The Construction of the Self: Developmental and Sociocultural Foundations.

[B39-jintelligence-13-00048] Keefer Kateryna, Holden Ronald, Parker James (2013). Longitudinal assessment of trait emotional intelligence: Measurement invariance and construct continuity from late childhood to adolescence. Psychological Assessment.

[B40-jintelligence-13-00048] Kickbusch Ilona (2012). Learning for Well-Being: A Policy Priority for Children and Youth in Europe—A Process for Change.

[B41-jintelligence-13-00048] Leal Fátima (2010). O Impacto da Inteligência Emocional no Rendimento Académico e nas Dificuldades de Aprendizagem. [The Impact of Emotional Intelligence on Academic Performance and Learning Difficulties]. Master’s thesis.

[B42-jintelligence-13-00048] Mayer John D., Salovey Peter, Salovey Peter, Sluyter David (1997). What is Emotional Intelligence?. Emotional Development and Emotional Intelligence: Educational Implications.

[B43-jintelligence-13-00048] Meshkat Maryam, Nejati Reza (2017). Does Emotional Intelligence Depend on Gender? A Study on Undergraduate Students. Applied Psychological Research.

[B44-jintelligence-13-00048] Pereira Marisa D. G. (2022). Diferenças Individuais na Inteligência Emocional em Crianças e Jovens: Influência da Idade e Género. [Individual Differences in Emotional Intelligence in Children and Youth: Influence of Age and Gender]. Master’s thesis.

[B45-jintelligence-13-00048] Petrides Konstantinos V., Pita Ria, Kokkinaki Flora (2007). The Location of Trait Emotional Intelligence in Personality Factor Space. British Journal of Psychology.

[B46-jintelligence-13-00048] Pfeifer Jennifer H., Peake Shannon J. (2012). Self-Development: Integrating Cognitive, Socioemotional, and Neuroimaging Perspectives. Developmental Cognitive Neuroscience.

[B47-jintelligence-13-00048] Plake Barbara S., Impara James C. (1999). Supplement to the Thirteenth Mental Measurement Yearbook.

[B48-jintelligence-13-00048] Pólvora Ana (2017). Influência das Avaliações de Pais e Professores na Inteligência Emocional em Contexto Escolar. [Influence of Parental and Teacher Assessments on Emotional Intelligence in School Contexts]. Master’s thesis.

[B49-jintelligence-13-00048] Rebelo Nicole (2012). Estudo dos Perfis Atitudinais e Emocionais de Alunos do Ensino Básico Português: Caracterização em Função do Nível Escolar e do Sexo. [Study of Attitudinal and Emotional Profiles of Portuguese Basic Education Students: Characterization by School Level and Gender]. Master’s thesis.

[B50-jintelligence-13-00048] Singh Magdeline, Thapa Preshna (2023). A Study of Emotional Intelligence of Higher Secondary School Students in the District of Kalimpong. International Journal for Research in Education.

[B51-jintelligence-13-00048] Tabachnick Barbara G., Fidell Linda S. (2007). Using Multivariate Statistics.

[B52-jintelligence-13-00048] Vilia Paulo, Candeias Adelinda, Neto António, Franco Glória, Melo Madalena (2017). Academic Achievement in Physics-Chemistry: The Predictive Effect of Attitudes and Reasoning Abilities. Frontiers in Psychology.

[B53-jintelligence-13-00048] Vilia Paulo, Candeias Adelinda (2019). Attitude towards the discipline of physics-chemistry and school achievement: Revisiting factor structure to assess gender differences in Portuguese high-school students. International Journal of Science Education.

